# Evolution of Robustness and Cellular Stochasticity of Gene Expression

**DOI:** 10.1371/journal.pbio.1001578

**Published:** 2013-06-11

**Authors:** Steven A. Frank

**Affiliations:** Department of Ecology and Evolutionary Biology, University of California, Irvine, California, United States of America; Princeton University, United States of America

## Abstract

Gene expression varies widely in cells with the same genotype and environment [1],[2]. Predicting the patterns of stochastic cellular fluctuations remains an unsolved challenge. I propose that the degree to which varying cellular components combine to determine robust phenotypes may predict the amount of variability. Microbes provide excellent experimental models to analyze the relations between robust phenotypes and stochastic variability.

## Abstract 2

Recent measurements of gene expression show a lot of randomness between cells. Steven Frank proposes a theory in which robustness protects against the effects of randomness and so allows relaxed regulatory control and greater randomness to evolve.

*This essay is part of the Challenges Series: highlighting fundamental, unifying challenges in biology.*

## Phenotype by Averaging of Components

Some phenotypes arise by averaging the inputs from many components. For example, a cell may capture an external signal and store the information internally. If the half-life of the internally stored information is sufficiently long, then a cell can average the inputs over a period of time [3]. For a signal associated with a constant message but subject to short-term stochastic fluctuations, averaging yields a more precise estimate of the message. The idea is simply that a larger sample size of independent measurements provides a more precise estimate of the average value. Pooling information into a larger sample may also happen by combining different kinds of signals or by different cells combining information about their different spatial locations [3].

Frank [4] pointed out that averaging inputs is a very general way in which to reduce variability in the expression of phenotypes. The greater the number of independent inputs, the less the variance in the average of the inputs. With a larger sample, perturbations to any input or component have less consequence for the overall output of the system. Robustness may reasonably be defined as reduced sensitivity to perturbation. Thus, averaging over multiple component inputs provides a powerful mechanism to achieve robustness of phenotypic expression and system performance.

## System Robustness Increases Component Variability

I have emphasized that combining component inputs increases the robustness of the system by reducing the consequences of component variability. I now turn the problem around. How does an increase in system robustness affect the variability of components?

Suppose that multiple component inputs are averaged to obtain a system output. The more independent component inputs, the less each component input affects the average. Therefore, as the number of inputs increases, the system can tolerate greater component variability to achieve the same level of output variability [4]. In a typical case with *n* independent inputs, each input having a variance of σ^2^, the variance of the average is σ^2^/*n*. If we double the number of inputs, then the system can tolerate a doubling of the variance of each component and still achieve the same variability in output.

If we assume that natural selection is acting on the system outputs—the phenotypes—then increasing the number of inputs weakens the selective force acting on the variability of each component. As selection on the reliability of the components weakens, we may expect greater component variability. In general, any evolutionary change that increases the robustness of system output tends to reduce the selective pressure acting on the reliability and variability of the individual components of the system [5]. Reduced selective pressure on the components may often lead to an increase in component variability.

This idea leads to a prediction: the more protections a system has against the variability of the underlying components, the more stochastic variability there will be in the underlying components. The logic is so simple and inevitable that it must, in a sense, be true. But a true theory is not necessarily an interesting or important biological theory. The force may be weak and of little consequence. To study the problem empirically, we may compare a phenotype that arises by aggregation of few components versus a phenotype that arises by aggregation of many components. Microbes often provide good model systems in which to test simple theories about natural selection.

## Aggregation and Variability

Bacteria often secrete enzymes to digest complex extracellular resources [6]. In a well-mixed environment, the total extracellular enzyme level depends on the aggregate secretions of the population. It is the total enzyme level that determines the digestion rate experienced by each individual cell. For a given average secretion rate per cell over the population, the variability in secretion rate among cells does not affect the digestion rate of the individual cells. Thus the observed cellular stochasticity for a given genotype should be relatively high. By contrast, when a function is at the cellular level, natural selection should more strongly constrain variability, and the observed cellular stochasticity for a given genotype should be relatively low. For example, a particular cell's uptake rate of externally digested products depends on the number of surface receptors of that cell. Thus natural selection will tend to constrain cellular variability in receptor number.

**Figure 1:**
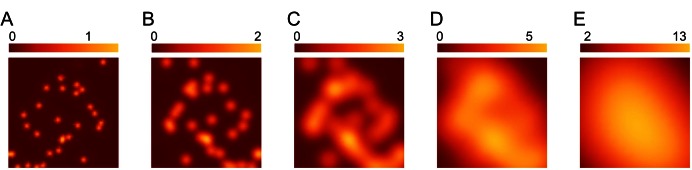
Averaging over cellular inputs by diffusion. Microbes use extracellular secretions for external digestion and other functions. The extracellular concentration of secretions at any location depends on the secretions of each cell in the population and the rate at which the secretions diffuse across space. When diffusion rates are low, then the concentration near a particular cell depends mainly on the secretion rate of that cell. When diffusion rates are high, then the concentration near a particular cell depends on the average secretion rate over all the cells in the common spatial neighborhood. (A) Concentration of an extracellular secretion in a low-diffusion environment. Each light-colored circle shows the location of a cell. The color index expresses the effective number of cells contributing to each spatial location. In this case, each cell is mainly affected by its own secretion rate. (B–E) Increasing diffusion rate causes a rise in the effective number of cells contributing to the extracellular concentration at each location. With high diffusion, the concentration depends on the average secretion rate over many different cells. If cells reduce their secretions in response to higher nearby extracellular concentration, then the total concentration may not change as the number of contributing cells per location increases. Instead, the same total may be achieved by a greater number of smaller inputs, in which case stochastic fluctuations in each input have less consequence for fluctuations in extracellular concentration. As the consequence declines for fluctuations of individual cells, one may expect weaker regulatory control per cell, and thus greater stochasticity per cell.

Several other microbial functions also happen extracellularly, such as quorum sensing and binding of free iron and vitamins by secreted molecules [7]. In well-mixed environments containing a clonal population, aggregate population expression determines the function of these traits. By contrast, in an environment with low extracellular diffusion rates, functional expression may occur at the level of each individual cell or a few neighboring cells (Figure 1). Thus, the rate of extracellular diffusion determines the level at which functional expression occurs and the strength of selection in constraining variability at a particular level. To test that idea, one could compare, for a particular trait, the variability in expression per cell in well-mixed versus limited-diffusion environments. One could also design experimental evolution studies in which one manipulated the extracellular diffusion rates and thus the level at which functional expression arises. Reduced diffusion should more strongly constrain variability at lower levels, because function aggregates over fewer components and thus natural selection on each component is stronger.

In multicellular organisms, some functions arise at the aggregate tissue level, whereas other functions depend on individual cellular expression. Cellular variability should be relatively high when function is at the aggregate tissue level compared to cases in which function is at the cellular level. Within cells, functional expression may arise by aggregation over the expression of several different gene products or pathways that effectively provide the same function. Redundant gene products or pathways are widely observed. Variability in redundant components is relatively weakly constrained and therefore is expected to be high.

## Aggregation and Robustness

Aggregation reduces the sensitivity of overall function with respect to the performance of each component. Thus natural selection may often favor a less costly, lower performance design of component traits [5]. Over time, the dynamics play out as follows. Aggregation and other robustness mechanisms reduce sensitivity to fluctuations of the components. Component traits, buffered by robustness mechanisms, degrade to lower performing and less costly states. Additional aggregation or robustness mechanisms arise. The cycle repeats. The multiple buffers of aggregation and robustness mechanisms become layered on top of each other, while the underlying traits become replaced by cheaper, lower performance components.

If one focused solely on the design and performance of the components over time, it would appear as if they were becoming increasingly maladapted. That apparent maladaptation arises because natural selection operates only on overall function rather than on the design and reliability of individual components. Clear interpretations of design in physiology and systems biology require consideration of components in relation to overall system function. Improving design at the system level can actually favor degradation of performance at the component level.

## Thought Experiment

It can be difficult to test the effects of a complex evolutionary sequence on the design of traits. Microbes may provide a good place to start. Consider a clonal microbial population faced with the challenge of digesting complex extracellular food sources. Initially, the population may be placed in a situation with low extracellular diffusion, so that each cell tends to be near only the digested products caused by its own enzymatic secretions (Figure 1A). Here, the system function, digestion rate per cell, depends directly on the secretions of the cell itself. After a period of selection under that situation, some of the resulting cells may be saved for later comparison.

Then a period of selection follows in a well-mixed environment. With mixture, each cell is near the digested products caused by the overall enzymatic secretions of the population (Figure 1E). The function, digestion rate per cell, depends only on the average secretion rate per cell in the population. In that case, the selective pressure against cellular variability in secretion rate is greatly reduced. In addition, a reduced cost of enzyme production and secretion may be favored, even if such cost reductions are associated with lower reliability or degraded performance of individual cells in the production of extracellular digestive enzymes. Because performance in a well-mixed environment depends only on the aggregate secretions of the clonal population, reduced component (individual) costs may lead to the most efficient aggregate function.

After a period of evolution in the well-mixed environment, the performance of individual cells may be compared to the cells derived from the initial period of selection in the low-diffusion environment. The prediction is that variability in expression will be greater in cells derived from the well-mixed environment. In addition, the overall performance and reliability of the cells from the well-mixed environment may be degraded relative to the cells from the low-diffusion environment.

To test for reduced costs of enzyme production in cells from the well-mixed environment, one may supplement the environment so that no benefit is derived from extracellular digestion but extracellular enzymatic secretion continues to be stimulated. In direct competition, the cells from the well-mixed environment may have an advantage relative to the cells from the spatially structured environment, because the well-mixed environment may have favored a reduction in the costs associated with the secretory traits.

## Prospects

There are, of course, many complexities of experimental design and interpretation in this scenario. For example, genetic variability may introduce competition between different genotypes, adding additional selective pressures with respect to the level at which function arises [7]. The thought experiment is meant only to clarify the concepts of aggregation, functional level, robustness, and the potential for apparent maladaptation in the design of components. These issues arise whenever function at a higher level develops by aggregation over lower level components. Other mechanisms of robustness besides aggregation may also have similar evolutionary consequences. Cleverly designed tests with microbes may provide a way to analyze these general aspects of system function and design.

With regard to the challenge of explaining patterns of stochastic variation in gene expression, the processes of aggregation and robustness provide intriguing hypotheses. Increased aggregation reduces the consequence of variability at the component level. Reduced selective pressure on the components may often lead to increased stochastic fluctuations of those components. Particular genes almost always play a component role within a larger functional system. The system structure by which component genes aggregate to determine function may be an important factor determining the amount of stochastic variation in gene expression.
